# Pulsed Electric Field (PEF) Enhances Iron Uptake by the Yeast *Saccharomyces cerevisiae*

**DOI:** 10.3390/biom11060850

**Published:** 2021-06-07

**Authors:** Karolina Nowosad, Monika Sujka, Urszula Pankiewicz, Damijan Miklavčič, Marta Arczewska

**Affiliations:** 1 Department of Analysis and Evaluation of Food Quality, Faculty of Food Sciences and Biotechnology, University of Life Sciences in Lublin, Skromna 8, 20-704 Lublin, Poland; karo.nowosad@gmail.com (K.N.); urszula.pankiewicz@up.lublin.pl (U.P.); 2 Faculty of Electrical Engineering, University of Ljubljana, Trzaska Cesta 25, 1000 Ljubljana, Slovenia; damijan.miklavcic@fe.uni-lj.si; 3 Department of Biophysics, Faculty of Environmental Biology, University of Life Sciences in Lublin, Akademicka 13, 20-950 Lublin, Poland; marta.arczewska@up.lublin.pl

**Keywords:** iron accumulation, yeast, iron deficiency, PEF

## Abstract

The aim of the study was to investigate the influence of a pulsed electric field (PEF) on the level of iron ion accumulation in *Saccharomyces cerevisiae* cells and to select PEF conditions optimal for the highest uptake of this element. Iron ions were accumulated most efficiently when their source was iron (III) nitrate. When the following conditions of PEF treatment were used: voltage 1500 V, pulse width 10 μs, treatment time 20 min, and a number of pulses 1200, accumulation of iron ions in the cells from a 20 h-culture reached a maximum value of 48.01 mg/g dry mass. Application of the optimal PEF conditions thus increased iron accumulation in cells by 157% as compared to the sample enriched with iron without PEF. The second derivative of the FTIR spectra of iron-loaded and -unloaded yeast cells allowed us to determine the functional groups which may be involved in metal ion binding. The exposure of cells to PEF treatment only slightly influenced the biomass and cell viability. However, iron-enriched yeast (both with or without PEF) showed lower fermentative activity than a control sample. Thus obtained yeast biomass containing a high amount of incorporated iron may serve as an alternative to pharmacological supplementation in the state of iron deficiency.

## 1. Introduction

Anemia affects about one-third of the world’s population; half of these cases are caused by iron deficiency. This is a serious and global public health problem that affects maternal and child mortality and their overall physical function. Children 0–5 years old, women of childbearing age and pregnancy are particularly at risk [1].

Iron is essential for numerous biological functions, including respiration, energy production, DNA synthesis and cell proliferation [2,3]. A sufficient supply of iron is necessary for the correct functioning of many biochemical processes, including electron transfer reactions, gene regulation, oxygen binding and transport, and regulation of cell growth and differentiation [4]. Iron deficiency is associated with chronic kidney disease, chronic heart failure, cancer and inflammatory bowel disease [5].

In addition to seeking and treating the cause of iron deficiency, treatment strategies include prevention, through iron supplementation and food enrichment [6]. Iron supplementation is necessary for high-risk groups (e.g., pregnant women). For oral supplementation, iron salts (ferrous sulfate and ferrous gluconate) are preferred because of their low cost and high bioavailability. Although iron absorption is increased when given on an empty stomach, nausea and epigastric pain may occur. Administration of iron during a meal reduces its absorption by approximately two-thirds [7]. Side effects of iron supplementation can be reduced by introducing iron-enriched foods into the diet [8]. Food products such as wheat flour, milk and infant formulas have been long used for iron fortification. Many countries have implemented programs of enrichment staple foods in iron, but they have disadvantages as some members of the population may not consume these products or have no regular access to these foods. Enriching food with iron is more difficult than with other nutrients such as iodine in salt and vitamin A in frying oil, because iron affects the sensory characteristics, changing the taste and color of the product. It can also cause fat oxidation [9]. To prevent these adverse changes, the microencapsulation process is used [10]. One of the encapsulation methods is the accumulation of minerals in the yeast cells of *S. cerevisiae* [11,12,13,14]. In the study of Kyyaly et al. [15] it was demonstrated that feeding anemic rats with iron-enriched yeast is more efficient than inorganic treatment in recovery from iron deficiency. In this case iron bioavailability from enriched yeast was higher than from ferrous sulfate heptahydrate.

Yeast is a single-cell organism that is used in brewing (beer and wine production), ethanol production, baking, and the production of recombinant proteins and biopharmaceuticals. Although there are many yeast species, *Saccharomyces* is by far the most commercially available [16]. Easily-grown and readily available yeasts such as baker’s or brewer’s strains of *S. cerevisiae* are excellent natural sources of essential metals such as K, Mg, Ca, Fe, Mn, and Zn and this yeast can be further enriched with other inorganic micronutrients (e.g., selenium) [17]. Metal accumulation in yeast involves a combination of extracellular accumulation and transport mechanisms. The first stage called biosorption is a reversible stage of accumulation. The second stage, usually referred to as active transport, is slower intracellular bioaccumulation, which is often irreversible and is associated with cellular metabolic activity [18]. The cell membrane is a barrier between the cell and its external environment. However, under special circumstances, the membrane barrier and transport selectivity can be partially lost, and molecules that do not pass through the intact cell membrane can get into the cell’s cytoplasm. This phenomenon occurs when cells are exposed to a pulsed electric field (PEF) [19].

The pulsed electric field (PEF) technique can be used as a non-thermal food preservation method that involves the use of short electrical pulses to reduce the microbial population while having minimal detrimental effects on food quality [20]. This process is also used to assist drying and can also cause stress reactions in plant systems or cell cultures [21]. Previous research has shown that this technique can be used for enriching *S. cerevisiae* yeast with metal ions [11,22,23]. Iron enriched yeast biomass can potentially be an additional source of this element in diet, especially for vegans and vegetarians.

The aim of the study was to investigate the influence of a pulsed electric field on the level of iron accumulation in *S. cerevisiae* cells. The ATR FTIR analysis was applied to determine the functional groups in yeast cells that could be involved in iron ions’ binding and demonstrate the effectiveness of PEF in enhancing the uptake of iron.

## 2. Results and Discussion

### 2.1. Selection of Iron Salt

**Figure 1:**
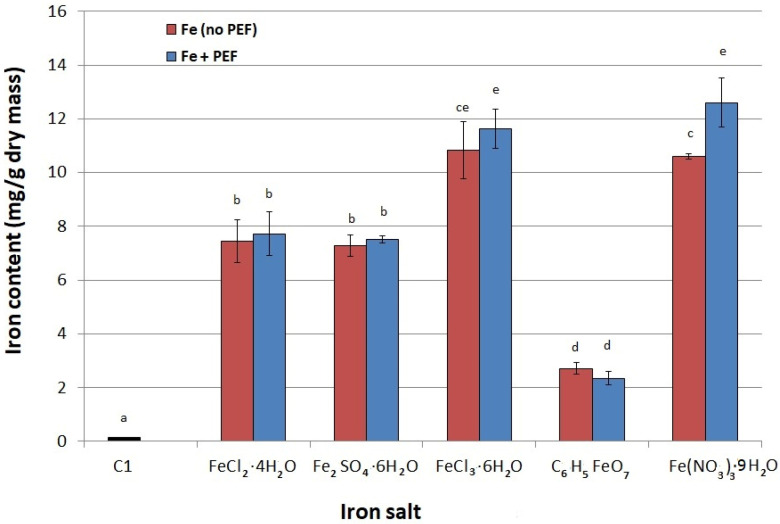
Effect of iron salt on iron accumulation in yeast cells: C1—control culture without iron ions added to the medium and PEF treatment; red bars—cultures not treated with PEF, blue bars—cultures treated with PEF (100 µg Fe/mL medium, voltage of 1500 V, pulse width of 10 µs, treatment time 10 min, number of pulses 600, after 20 h of cultivation). Each value is the mean ± standard deviation (*n* = 3). Bars with the same letter (a–e) are not significantly different (*p* < 0.05).

In the first stage of the experiment we selected the iron salt for which the highest accumulation of this element in yeast was observed. Cells derived from the culture not supplemented with iron and not exposed to PEF contained only 0.13 mg Fe/g dry mass and were used as control (C1). This level of iron accumulation is in agreement with that reported by Zachariadis et al. [24] for active dry yeast. In our experiments each iron salt was added to two cultures and one of them was additionally treated with PEF. Studies have shown that the greatest effect of PEF on iron accumulation in *S. cerevisiae* cells occurred when iron (III) nitrate was used as the source of iron ions (Figure 1). This may be related to the fact that iron (III) nitrate is derived from a strong acid and is likely to be in a dissociated form, thus providing free iron for binding to cells [25]. In the case of this salt, the iron concentration in yeast cells was almost 97 times higher in the PEF-treated sample than in the control sample with no iron salt added and not subjected to PEF (C2). For the remaining salts, no significant effect of PEF on iron accumulation was observed. Generally, yeast accumulated the least amount of iron from iron citrate, which may be due to the change in pH of the medium from 5.0 to about 4.4. Lower pH may cause incomplete dissociation of iron citrate, which explains the lower intracellular iron content compared to other salts [26]. Additionally, some authors have observed a toxic effect of iron (III) citrate on yeast cells [27,28]. According to Paš et al. [28] this effect can be caused not only by iron, but also by the anionic part of this iron compound.

**Figure 2:**
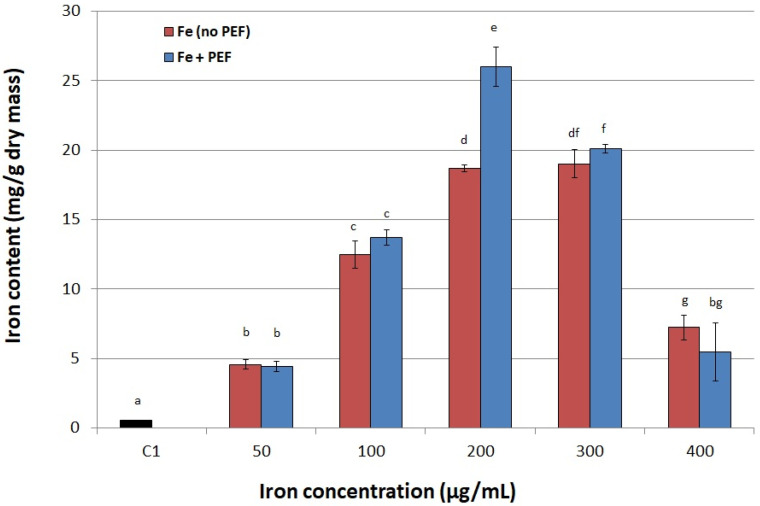
Effect of iron concentration on its accumulation in yeast cells. C1—control culture without iron ions added to the medium and PEF treatment; red bars—cultures not treated with PEF, blue bars—cultures treated with PEF (ferric nitrate, voltage of 1500 V, pulse width of 10 µs, treatment time 10 min, number of pulses 600, after 20 h of cultivation). Each value is the mean ± standard deviation (*n* = 3). Bars with the same letter (a–g) are not significantly different (*p* < 0.05).

Our experiments showed that at low concentrations of iron ions in the culture medium (in the range of 50–100 µg/mL) PEF treatment had no effect on iron accumulation in yeast. Statistically significant changes were noted at 200 µg Fe^3+^/mL, and then the difference in accumulation between cells supplemented with iron without PEF treatment and PEF-treated cells was the largest and amounted to 8.23 mg/g dry mass. We observed that iron uptake was reduced when concentration was higher than 200 µg Fe^3+^/mL (Figure 2), so we adopted the concentration of 200 µg Fe^3+^/mL as optimal for effective accumulation of this element in further experiments. High iron ions concentration in the medium may cause precipitation reactions, which may be due to the formation of iron hydroxides, their polymerization or the formation of poorly soluble phosphate iron [28]. Iron is an essential ingredient for yeast, but its high content in the culture medium can also be toxic. For this reason the uptake and utilization of iron in yeast cells is tightly regulated [29]. *S. cerevisiae* can grow in environments with both too little and too much iron [30]. A study by Philpot and Protchenko [25] indicates that when iron is restricted, cells will not only increase iron uptake, but also adjust their metabolism to use the available iron more efficiently.

### 2.2. Conditions of PEF Treatment

**Figure 3:**
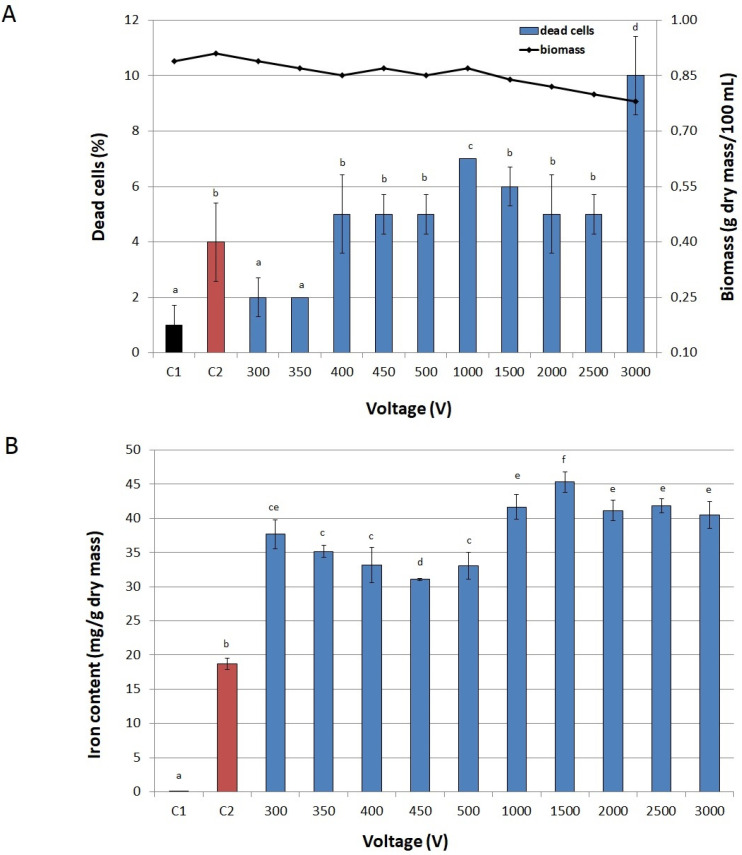
Effect of voltage on viability of cells and biomass (**A**) and on iron accumulation in yeast cells (**B**). C1—control culture without iron ions added to the medium and PEF treatment, C2—control culture with iron ions added to the medium (200 μg/mL) and without PEF treatment, blue bars—cultures treated with PEF (ferric nitrate, 200 μg Fe^3+^/mL, pulse width of 10 µs, treatment time 10 min, number of pulses 600, after 20 h of cultivation). Each value is the mean ± standard deviation (*n* = 3). Bars with the same letter (a–f) are not significantly different (*p* < 0.05).

**Figure 4:**
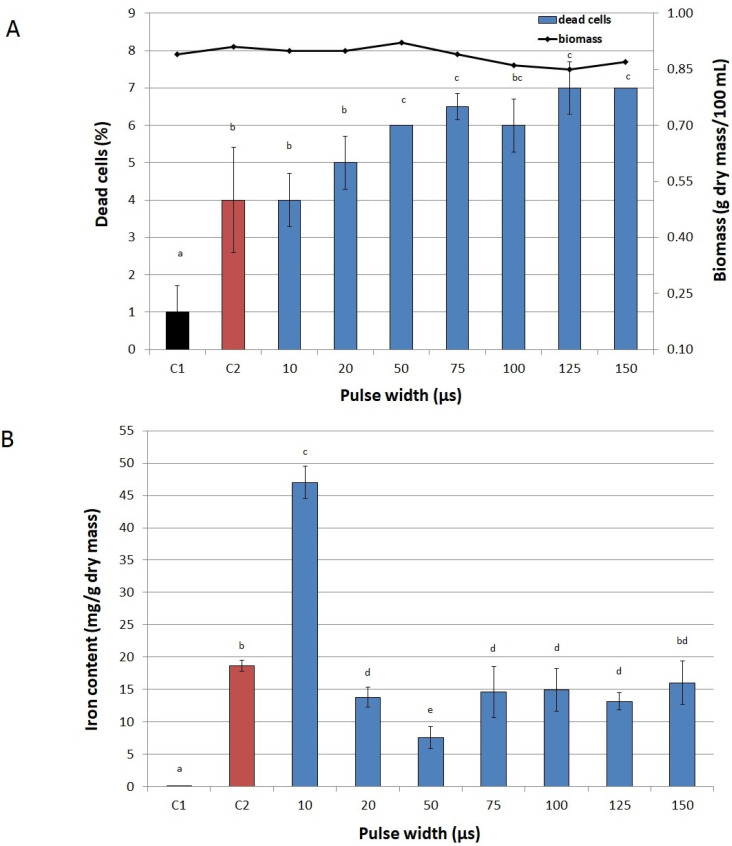
Effect of pulse width on viability of cells and biomass (**A**) and on iron accumulation in yeast cells (**B**). C1—control culture without iron ions added to the medium and PEF treatment, C2—control culture with iron ions added to the medium (200 μg/mL) and without PEF treatment, blue bars—cultures treated with PEF (ferric nitrate, 200 μg Fe^3+^/mL, voltage 1500 V, treatment time 10 min, number of pulses 600, after 20 h of cultivation). The pulse width was varied from 10 µs to 150 µs at 1500 V voltage amplitude. Each value is the mean ± standard deviation (*n* = 3). Bars with the same letter (a–d) are not significantly different (*p* < 0.05).

**Figure 5:**
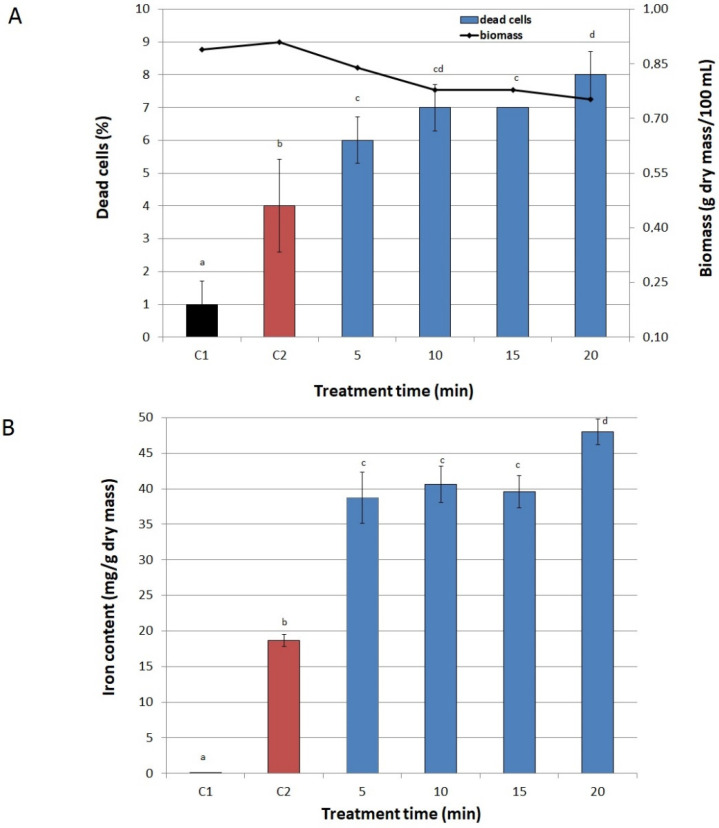
Effect of treatment time on viability of cells and biomass (**A**) and on iron accumulation in yeast cells (**B**). C1—control culture without iron ions added to the medium and PEF treatment, C2—control culture with iron ions added to the medium (200 μg/mL) and without PEF treatment, blue bars—cultures treated with PEF (ferric nitrate, 200 μg Fe^3+^/mL, voltage 1500 V, pulse width of 10 µs, after 20 h of cultivation). Each value is the mean ± standard deviation (*n* = 3). Bars with the same letter (a–d) are not significantly different (*p* < 0.05).

Figure 3, Figure 4 and Figure 5 present the effect of PEF parameters on biomass production and cell viability, as well as on iron accumulation in cells. Only slight fluctuations in the number of inactivated cells in the entire range of tested values of voltage were observed. The highest fraction of dead cells in a culture (10%) was observed at 3000 V (Figure 3A). A significant drop in biomass production (from 0.87 to 0.78 g dry mass/100 mL) was noted only at voltages higher than 2000 V. In the case of pulse width, we observed a significant decrease in biomass in the range from 75 μs to 150 μs at 1500 V. The pulse width also influenced the viability of yeast cells (Figure 4A). At 10 µs it was the same as in the control not exposed to PEF (C2), however, for values higher than 50 µs, cell viability began to decline. But still we noted high values for both parameters—the share of dead cells did not exceed 10% and biomass was over 0.7 g dry mass/100 mL. Similarly, Stirke et al. [31] did not observe a significant impact of pulse durations of less than 100 μs on yeast cells’ viability. Treatment time had little effect on biomass and cell viability (Figure 5A). Although the biomass production dropped significantly already after 5 min treatment, compared to the control cultures, this decrease was not sharp. After 20 min of treatment the biomass production was 15% lower than in the control sample not exposed to PEF, but the total number of inactivated cells was rather low (only 8%). Longer treatment resulted in a higher decrease in biomass and cells viability.

Temperature is also a critical parameter that influences the efficacy of PEF treatment [32]. In our experiments the temperature was continuously monitored and was between 24 and 26 °C. There were no drastic changes that could influence the conductivity of the medium or cell growth.

Application of low values of voltage (300–500 V) resulted in a twofold increase in iron content in cells compared to the control sample not exposed to PEF and with iron added to the medium (Figure 3B). The highest iron accumulation (over 2.6 times higher than in the abovementioned control sample) was achieved at 1500 V. Higher values of voltage caused a significant decrease in iron content in yeast cells, which can be related to a small decrease in biomass and yeast viability. The highest concentration of iron in cells was recorded at the pulse width of 10 μs (Figure 4B), similarly to Pankiewicz and Jamroz [23], who also observed the highest accumulation of zinc in *S. cerevisiae* cells (15 mg/g dry mass) after PEF treatment with a pulse width of 10 μs. The concentration of iron in the cells exposed to PEF at 20, 50 and 75 μs, as well as at 100, 125 and 150 μs did not differ significantly.

Studies on the treatment time were carried out in the range of 5–20 min. Iron concentration in *S. cerevisiae* cells increased with increasing time, reaching the maximum (48.01 mg/g dry mass) at 20 min (Figure 5B). Pankiewicz and Jamroz [23] reported that the accumulation of zinc in *S. cerevisiae* cells was the highest after 15 min of PEF treatment. The difference in treatment time between our study and the studies by Pankiewicz and Jamroz [23] may be caused by the incorporation of different element into the cells of *S. cerevisiae*.

In recent years many articles on electroporation have been published using, among others, the yeast *S. cerevisiae* as a model organism. Stirke et al. [31,33] investigated the absorption of the tetraphenylphosphonium (TPP+) ion by the yeast *S. cerevisiae*. They applied electric field pulses with a duration from 5 to 150 μs and amplitude up to 10 kV/cm. The obtained results confirmed that for pulses with a duration less than 100 μs, the permeabilization is increased and no significant decrease of cell viability is observed, which is in agreement with our findings.

**Figure 6:**
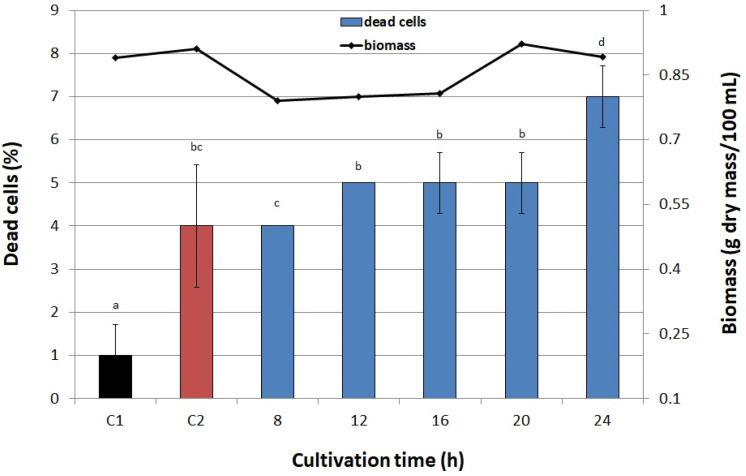
Effect of cultivation time after which PEF was applied on viability of cells and biomass. C1—control culture without iron ions added to the medium and PEF treatment, C2—control culture with iron ions added to the medium (200 μg/mL) and without PEF treatment, blue bars—cultures treated with PEF (ferric nitrate, 200 μg Fe^3+^/mL, voltage 1500 V, pulse width 10 μs, treatment time 20 min, 1200 pulses). Each value is the mean ± standard deviation (*n* = 3). Bars with the same letter (a–d) are not significantly different (*p* < 0.05).

Also the assumption that the influence of the PEF on the yeast cell wall increases its permeability to TPP+ was confirmed. This process can be controlled by appropriately setting the amplitude and duration of the PEF. The authors also concluded that the similar characteristic lifetimes of the non-equilibrium pores in the cell wall and membrane after PEF treatment indicate a strong coupling between these parts of the cell. Experiments carried out on Chinese hamster lung fibroblast cells (DC-3F) and human adipose mesenchymal stem cells (haMSC) in the medium containing Ca^2+^ using microsecond pulsed electric fields confirmed the influx of extracellular calcium induced by the electric pulse as a result of the electropermeabilization of the cell membrane [34]. The final step in our experiment was setting the cultivation time after which yeast cells were exposed to PEF. We considered the optimal time after which the highest biomass production was achieved. In our case it was 20 h (0.92 g dry mass/100 mL) (Figure 6), similarly to Pankiewicz et al. [12] and Pankiewicz and Jamroz [14], who treated *S. cerevisiae* cultures with PEF to improve, respectively, the simultaneous accumulation of magnesium and zinc, and accumulation of selenium in biomass. However, there are considerable discrepancies in the optimal time of cultivation set in studies on the accumulation of iron from the medium by yeasts, which may result from different culture conditions. For instance, Stehlik-Thomas et al. [35] reported that the highest concentration of iron in cells (10 mg/g dry yeast biomass) was obtained after 12 h of cultivation in anaerobic conditions. On the other hand, under semiaerobic conditions, the highest accumulation was achieved after 16 h of cultivation, but it was four times lower (2.5 mg/g dry yeast biomass) than the above-mentioned one. Wang et al. [36], in turn, obtained the highest Fe content (7.854 mg/g dry mass) in yeast cultivated for 60 h at 30 °C.

### 2.3. Fluorescence Imaging of Yeast Cells

**Figure 7:**
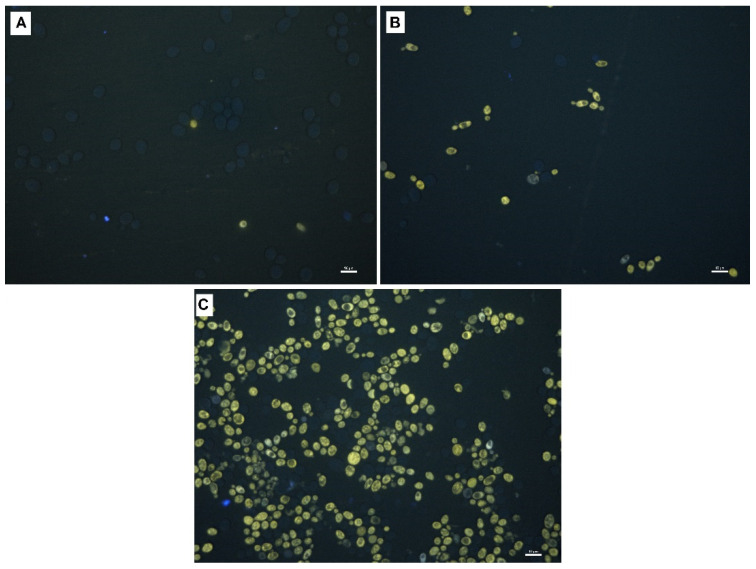
Fluorescent microscopy images of yeasts stained with Rhodamine B: (**A**) C1—control culture without iron ions added to the medium and PEF treatment, (**B**) C2—control culture with iron ions added to the medium (200 μg/mL) and without PEF treatment, (**C**) culture with iron added to the medium (200 μg/mL) and PEF treatment (ferric nitrate, voltage 1500 V, pulse width 10 µs, treatment time 20 min, 1200 pulses, after 20 h of cultivation). The scale bars correspond to 10 μm.

Figure 7 presents the images of yeast cells stained with Rhodamine B and observed under a fluorescence microscope. This dye crosses cell membranes and is captured by mitochondria without inducing cell lysis. Rhodamine-based dyes can be used to bioimage iron pools in living cells [37]. Almost all cells from the control sample not subjected to PEF and without iron supplementation were dark (Figure 7A), whereas those from the sample enriched with iron without PEF partly showed green fluorescence (Figure 7B). PEF treatment enhanced the accumulation of iron in yeast cells, so almost the entire population of cells showed strong green fluorescence (Figure 7C). The detected fluorescence emission is caused by the iron complexation-induced opening of the spirocyclic ring of the rhodamine-based probes [37] and its relative intensity is proportional to the iron concentration [38]. In our previous studies [39] we have observed yeast cells enriched with calcium and zinc by PEF treatment and stained with, respectively, calcium orange and morin under a confocal laser microscope. The experiments were carried out with the same PEF treatment system as this used here. We have done semi-quantitative analysis of ions within the limits of the cells. The study revealed that that fluorescence inside cells from control samples (without ions added to the medium and PEF treatment) was lower than that observed for cells from the sample enriched with calcium and zinc using PEF. On the basis of the optical sections, we also have made the 3D reconstructions of ion-rich areas distribution in the cell. It has been confirmed that in PEF-treated yeast cells absorption of calcium and zinc ions was higher and that metal ions were distributed inside the cell.

### 2.4. Attenuated Total Reflectance-Fourier Transform Infrared Spectroscopy

FTIR spectroscopy is a non-destructive and an excellent tool for rapid studying of a fingerprint of the biological samples under investigation, giving information on its most important biochemical components such as proteins, lipids, nucleic acids, and carbohydrates, cell wall and membranes. The ATR-FTIR spectra of lyophilized yeast cells stained from the control sample (C1), the iron-enriched samples both without PEF treatment (C2) and with the use of PEF at optimal conditions (Fe + PEF) were recorded in the region between 4000 and 750 cm^−1^. The location of the characteristic bands corresponds to the data reported in the literature [40,41,42,43,44], (Figure S1, Table S1).

**Figure 8:**
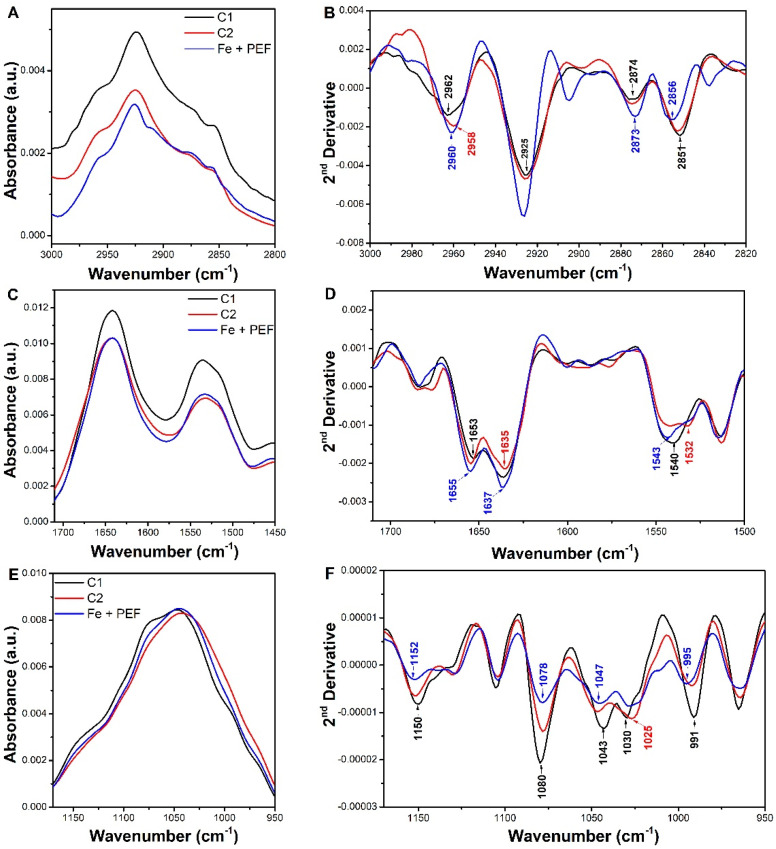
The ATR FTIR spectra of yeasts stained and their second derivatives in the selected ranges. (**A**,**B**): the region of the vibrational modes mainly due to the asymmetric and symmetric stretching vibrations of CH_2_ and CH_3_ methylene groups (**C**,**D**): the region of amide I and amide II bands; (**E**,**F**): the region of carbohydrates modes. The second derivative spectra have been normalized to the CH_2_ band at ~2924 cm^−1^ (**B**), to the amide I (**D**) while in panel F spectra have been normalized at the area under range 1180-900 cm^−1^. C1-control culture without iron added to the medium and PEF treatment (black line), C2-control culture with iron ions added to the medium (200 μg/mL) and without PEF treatment (red line), and Fe + PEF culture with iron ions added to the medium (200 μg/mL) and treated with PEF (ferric nitrate, voltage 1500 V, pulse width 10 µs, treatment time 20 min, 1200 pulses, after 20 h of cultivation) (blue line).

The broad peak at about 3290 cm^−1^ indicated the presence of both amine (N–H) and bonded hydroxyl (O–H) groups (Figure S1). The lipid region between 2800 and 3000 cm^−1^ is dominated by the C–H symmetric and asymmetric stretching vibrations of the CH_2_ and CH_3_ groups assigned to fatty acid chains in phospholipid membranes and the cell wall (Figure 8A). The spectra between 1700 and 1500 cm^−1^ are dominated by the amide I and amide II bands, respectively due to the C=O stretching and the N–H bending of the peptide bond (Figure 8C). The amide I band particularly provides information on the protein secondary structure due to the decomposition in several subbands characteristic of the different protein conformations: α-helix (~1656 cm^−1^), and β-sheet (1627–1635 cm^−1^) [45]. The bands within the region of 1200–900 cm^−1^ are associated with the mixed vibrations of polysaccharides (C–O–H and C–O–C of the mannan and glucan vibrations), nucleic acids (P=O), and lipids (C-H bending modes) [41,46] (Figure 8E). To better estimate possible spectral changes due to iron accumulation in yeast cells, the second order derivative spectra have been generated to resolve the overlapping bands into individual ones, thus increasing the accuracy [47]. The predominant bands acquired in second derivative spectra of examined samples and their attribution to specific chemical groups are summarized in Table 1. The second derivative spectra of yeast cells from the C1, C2 and Fe + PEF samples were determined in the three significant ranges, which are dominated by bands associated with the absorption modes of lipids, proteins and carbohydrates (Figure 8B,D,F).

As can be noted, the changes in the spectral features of the lipid region (3000–2800 cm^−1^) are related to a substantial increase in intensity of the CH_2_ bands at 2925 cm^−1^ in case of the samples subjected to PEF (Figure 8B). Moreover, a weaker increase in the intensity of the CH_3_ absorption at ~2960 and ~2873 cm^−1^ has been also detected for both the culture iron-supplemented samples. The variation of the CH_2_ and CH_3_ contribution could be related to the inducing change in yeast membrane fluidity by the means of iron ions modifications. In addition, a 1–4 cm^−1^ upward shift of the CH_2_ asymmetric and symmetric stretching bands was observed especially in yeast subjected to PEF (Figure 8B). Indeed, the state of lipid cell membranes are related to the spectral shift of the CH_2_ bands into lower or higher frequencies and correspond, respectively, to their rigidity or fluidity [47] Ganeva et al. [48] and Strike et al. [31] have postulated that yeast treatment with PEF could induce not only the membrane permeabilization, but also cause the changes in the structure of cell wall, leading to enhance the yeast cell wall porosity.

When the yeast cells from the control culture and its metal modified species are compared, the second derivative profiles of amide I and II are quite similar except for the position of the bands (Figure 8D). Indeed, the peak position at 1653 cm^−1^ (assigned to α-helix) and 1635 cm^−1^ (assigned to β-sheets and most likely intracellular water) are affected both in the case of C2 and Fe + PEF which was seen in marginal frequency shifts, and variations in absorbance intensities, compared with yeast from the control culture C1. This shifting of the amide I band to 1655 cm^−1^ was related to the involvement of the O and N atoms of the polypeptide chain in iron ions’ binding (Figure 8D).

The changes of a broad peak shape in the region 1180–950 cm^−1^ for metal-modified yeast in comparison to the control yeast sample may indicate the interactions of iron ions with polysaccharides present in the yeast cell wall. Indeed, the majority of polysaccharides of the yeast cell is found in its wall [49]. More specifically, the inner wall layer consists of mainly β-1,3-glucan but the outer one is formed by highly glycosylated mannoproteins with numerous phosphate groups in their carbohydrate side chains, resulting in a net negative surface charge. In fact, in the second derivative spectra of yeast after PEF treatment, a band attributed to β-1,3-glucan (1150 cm^−1^) is much less intense, broadened and shifted to a higher frequency suggesting its participation in iron ions’ binding. Taking into account other carbohydrate-related bands, a decrease in intensity of the bands assigned to the glucan structure, namely, at 1080 cm^−1^, 1043 cm^−1^ (mannans), 1030 and 991 cm^−1^ (β-1,6 glucans), and accompanied with their spectral shift compared to control C1 was also observed (Figure 8F). Finally, a strong reduction of the band at about 1080 cm^−1^ observed in the yeast sample after PEF treatment may suggest the interaction of the PO_2_^−^ of membrane phospholipids with the positive iron ions. The reduction of the abovementioned band intensities can be explained by iron ions binding to yeast cells as a result of presence of bond stretching to a lesser degree [50], so the iron ions could pass through the cell wall and periplasmic space and reach the surface of the plasma membrane to a large extend by PEF treatment.

### 2.5. Fermentative Properties and Protein Content in Yeast Cells

**Figure 9:**
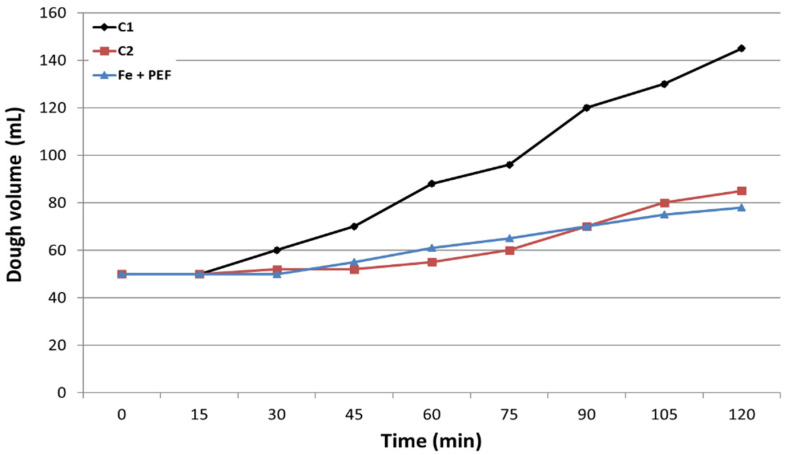
Effect of PEF and iron enrichment on fermentative properties of yeast: C1—control culture without iron added to the medium and PEF treatment, C2—control culture with iron ions added to the medium (200 μg/mL) and without PEF treatment, Fe + PEF—culture with iron ions added to the medium (200 μg/mL) and treated with PEF (ferric nitrate, voltage 1500 V, pulse width 10 µs, treatment time 20 min, 1200 pulses, after 20 h of cultivation). Each value is the mean ± standard deviation (*n* = 3).

In this study we also investigated the effect of PEF on the fermentation properties of yeast. Figure 9 shows the results obtained for the controls C1 and C2, and for the sample obtained at the optimal PEF conditions (Fe + PEF). The control C1 showed the highest fermentative activity. Already in the 30th minute of the test, dough growth was observed, while the dough with the addition of yeast with C2 and Fe + PEF started to rise only after 60 min. After 120 min the volume of dough containing yeast from the control C1 was 1.7-fold higher than that with yeast from the control C2 and 1.85-fold higher than in the case of dough with yeast exposed to PEF. The decrease in fermentative activity of the control C2 and the PEF-exposed sample (Fe + PEF) compared to C1 can be partly explained by their lower content of protein (Table 2). Another factor influencing yeast fermentation is the availability of assimilable nitrogen. Generally, *S. cerevisiae* yeasts are unable to use nitrate as sole nitrogen source [51] because, being deprived of molybdenum-dependent enzymes, they cannot assimilate it [52]. 

The study of Święciło [53] also demonstrated that *S. cerevisiae* show low sensitivity to sodium nitrate (V) which was explained by the fact that nitrates (V) are removed from cells efficiently. The author observed a 50% death rate of the yeast population when the concentration of this salt was as high as about 1 mol/L. The lower fermentation performance of yeasts from the sample C2 and that treated with PEF may also be related to the presence of high concentration of iron in cells. Iron, along with potassium, magnesium, calcium, manganese, copper and zinc, is one of the most important metals that influence yeast fermentation processes [17]. Excess iron can be detrimental to cells as certain forms can be involved in Fenton redox reactions that accelerate the formation of reactive oxygen species (ROS), such as hydroxyl radicals that damage cells at the level of membranes, proteins and nucleic acids [54]. Oxidative damage to proteins is expected to have negative consequences on the fermentative ability of yeast [55].

## 3. Materials and Methods

### 3.1. Microorganism and Growth Media

The industrial strain of *S. cerevisiae* 11 B1 from the Yeast Plant (Lublin, Poland) was used. The composition of medium for agar slants and inoculum growth, as well as that used in the experiment with PEF is presented in Table 3. The pH was adjusted to 5. All reagents were of analytical grade purity.

### 3.2. Biomass Cultivation

The yeast was passaged three times on agar slants, grown for 48 h in a thermostat at 30 °C, and finally used for the inoculum preparation. Cells from one slant were used to inoculate 150 mL of sterile medium in an Erlenmeyer flask. Cultures were grown in a shaking incubator (NBB 205L, N-BIOTEK Inc., Gyeonggi-Do, Korea) at 30 °C and 100 rpm for 48 h.

The culture medium was centrifuged after 48 h of culture and the cell pellet was washed three times with sterile water. The pellets from three Erlenmeyer flasks were collected and resuspended in sterile water to a final volume of 300 mL. 10 mL of the thus prepared inoculum was used to inoculate the immersion cultures into 500 mL Erlenmeyer flasks, each containing 90 mL of medium. Growth conditions were identical to the inoculum.

### 3.3. Design of Experiments for Optimization Iron Accumulation

Process parameters were optimized by maintaining all factors at a constant level except the one under study (one-factor-at-a-time (OFAT) method). The selection of parameter values was based on the previous studies by Pankiewicz and Jamroz [11]. The following parameters were optimized: iron concentration, voltage, pulse width, treatment time and cultivation time. Iron salt was selected in a separate experiment.

### 3.4. PEF Treatment

**Figure 10:**
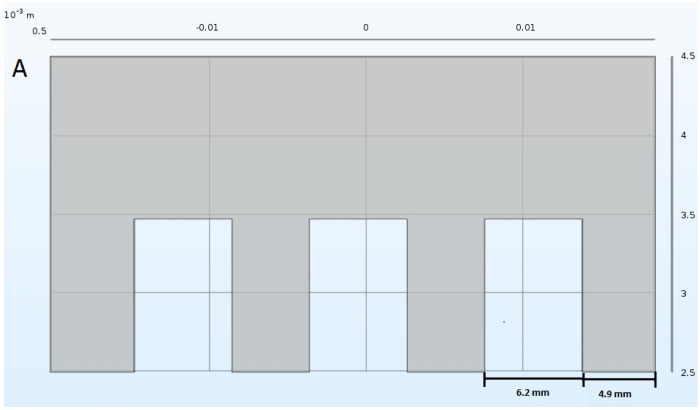

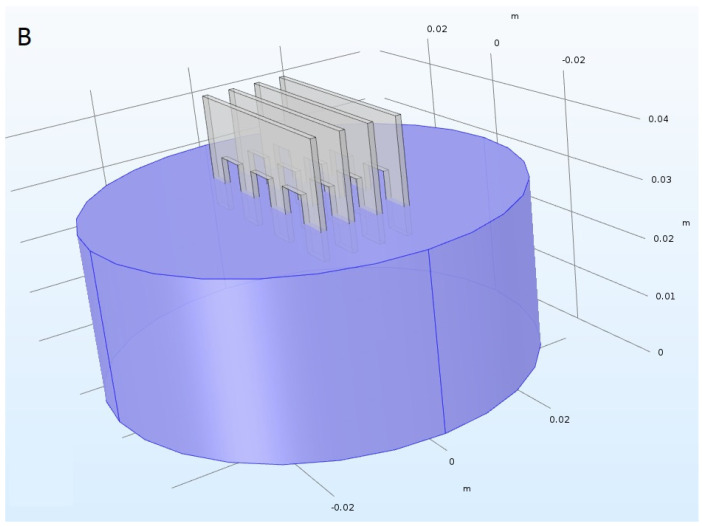
Schematic representation of a single electrode (**A**) and set of electrodes immersed into the culture medium (**B**).

The cultures of *S. cerevisiae* were grown in flasks under continuous agitation for 20 h and then treated with PEF using an ECM 830 unipolar square wave generator (BTX Harvard Apparatus, Holliston, MA, USA). A culture with a volume of 100 mL was placed in the PEF treatment chamber consisted in a beaker (300 mL) and four parallel stainless steel electrodes of an area equal to 4 cm^2^, opposed to each other with a spacing of 5.1 mm (Figure 10), mounted on a removable cover. The conductivity of the culture medium was 2.6 mS/cm (conductometer CC-505, Elmetron, Zabrze, Poland) and the frequency for delivering pulses was 1 Hz. While delivering the pulses the solution was being mixed with the help of a rotating magnet (100 rpm) to avoid cells sedimentation. The electrodes were immersed for approximately 7.4 mm into the solution. The temperature was monitored during PEF treatment.

### 3.5. Selection of Iron Salt

In order to select the iron salt with the highest accumulation in yeast cells five iron salts (FeCl_2_•4H_2_O, Fe_2_SO_4_•6H_2_O, FeCl_3_•6H_2_O, Fe(NO_3_)_3_•9H_2_O, and iron (III) citrate) were added to the medium in concentration of 100 µg Fe/mL. The conductivity of the solution was 2.7 mS/cm. Cultures after 20 h cultivation were exposed to PEF with the following initial conditions: voltage of 1500 V, pulse width of 10 µs, treatment time 10 min, number of pulses 600. Then cultures were centrifuged, washed several times with deionized water, and lyophilized in a Model 64132 freeze dryer (Labconco, Kansas City, MO, USA). At the same time, a control sample (C1) without iron in the medium and PEF treatment was prepared.

### 3.6. Selecting Optimal Process Parameters

#### 3.6.1. Iron Concentration in the Medium

The freshly prepared solution of selected iron salt was added to the medium just before electroporation so that the concentration of iron ions in a sample was, respectively, 100, 200, 300, and 400 µg/mL and conductivity of the solutions was in the range 2.7–3.0 mS/cm, respectively. Then the culture was subjected to PEF with the same parameters as described in Section 3.4. Simultaneously a control sample C1 was prepared.

#### 3.6.2. Voltage

After selecting the iron concentration, voltage was screened in the range 300–3000 V at the constant pulse width of 10 µs, treatment time of 10 min, and number of pulses 600. Two untreated samples—one with no iron ions in the medium (C1) and the other with 200 µg Fe^3+^/mL (C2), served as controls.

#### 3.6.3. Pulse Width

Pulse width was tested for the values of 10, 20, 50, 75, 100, 125 and 150 µs at the constant voltage of 1500 V, treatment time of 10 min, and number of pulses 600. Two untreated samples—one with no iron ions in the medium (C1) and the other with 200 µg Fe^3+^/mL (C2), served as controls.

#### 3.6.4. Treatment Time

In the final step, treatment time was varied for 5, 10, 15, and 20 min with constant values of other parameters. During the treatment time pulses were delivered at 1 Hz pulse repetition frequency. Two untreated samples—one with no iron ions in the medium (C1) and the other with 200 µg Fe^3+^/mL (C2), served as controls.

#### 3.6.5. Cultivation Time

Cultures were subjected to PEF after 8, 12, 16, 20, and 24 h of cultivation. The following PEF parameters were applied: voltage of 1500 V, pulse width 10 µs, treatment time 20 min, 1200 pulses. Two untreated samples—one with no iron ions in the medium (C1) and the other with 200 µg Fe^3+^/mL (C2), served as controls.

After each stage of experiment cells were centrifuged, washed several times with deionized water, and then lyophilized in a Model 64132 Labconco freeze dryer. The experiment was performed in triplicate.

### 3.7. Determination of Iron Concentration

Iron concentration was determined using the flame atomic absorption spectrophotometry (FAAS, Solaar 939, Unicam, Cambridge, UK). Samples of the freeze-dried biomass were weighed into the thimbles, flooded with 3 mL of HNO_3_–HClO_4_ (5:1) mixture and mineralized for 20 min at 250 °C in a microwave oven (MARS 5, CEM Corporation, Matthews, NC, USA). After cooling, solutions were transferred to 10 mL measuring flasks and topped up with deionized water [11]. The determination was performed in triplicate.

### 3.8. Determination of Yeast Biomass and Cell Viability

Cell biomass was estimated by measurement of optical density at 600 nm against pure culture medium in 2-mm measurement cells. Then dry mass was calculated using equation for the standard curve. In the case of cell viability, dead cells were counted in the Thoma chamber after staining with the 0.01% methylene blue solution. Both determinations were performed in triplicate.

### 3.9. Determination of Fermentative Properties and Protein Content in Yeast Cells

Fermentative activity of yeast was determined by measuring the volume of the expanding dough over time (0–120 min) [56]. Protein content in yeast cells was determined by the Kjeldahl method [57]. The results are means of three measurements.

### 3.10. Fluorescent Microscopy

Yeast cells from the samples C1 and C2, and the sample enriched with iron at the optimal PEF conditions were stained with an 0.01% ethanol solution of Rhodamine B (Merck, KGaA, Darmstadt, Germany) and observed under a fluorescent microscope (Eclipse 90i, Nikon, Tokyo, Japan). The excitation and emission wavelengths were 550 nm and 580 nm, respectively.

### 3.11. Attenuated Total Reflectance-Fourier Transform Infrared Spectroscopy

Mid-infrared absorption spectra were acquired by Attenuated Total Reflectance-Fourier Transform Infrared spectroscopy (ATR-FTIR) IRSpirit (Shimadzu, Kyoto, Japan) equipped with a DLATGS detector. The measurements were performed in attenuated total reflectance mode using the QATR™-S Single-Reflection ATR Accessory with a Diamond Crystal (Shimadzu). A pinch of dried samples were placed directly onto the crystal (with a contact area diameter of 1.8 mm) and pressured against its surface with a swing clamp mechanism. Spectra were collected with 36 spectral scans at a resolution of 4 cm^−1^ within the wavenumber range between 4000 and 500 cm^−1^. The spectra were ATR, air vapour and baseline corrected and normalized. The spectral normalization was performed in terms of the equal area in the appropriate spectral range (3000–2800 cm^−1^ for lipids, 1720–1500 cm^−1^ for proteins, 1180–900 cm^−1^ for carbohydrates). To gain more insight into cell structural components, second derivation procedure was performed. All spectral and data analysis were performed using the Grams/AI 8.0 software (Thermo Scientific, Waltham, MA, USA).

### 3.12. Data Analysis

Regression analysis and significance tests were performed using the Statistica 13.3. software (StatSoft, Inc., Tulsa, OK, USA). The pos-hoc Tuckey test was employed to determine differences between means. Results of *p* < 0.05 were considered statistically significant.

## 4. Conclusions

This study showed that pulsed electric field increases iron accumulation in *S. cerevisiae* cells. At the iron ion concentration of 200 µg/mL and under the following PEF conditions: voltage of 1500 V, pulse width of 10 μs, treatment time 20 min, number of pulses 1200, as well as the optimal cultivation time of 20 h, the amount of accumulated iron increased from 18.68 mg/g dry mass (for the control culture supplemented with iron but not treated with PEF) to 48.01 mg/g dry mass. At the same time, treatment with PEF did not significantly influence biomass production and cell viability. FTIR analysis of unloaded and iron ions loaded yeast cells in the range of 4000–900 cm^−1^ allowed us to determine the presence of functional groups that could be involved in iron ion binding. Yeast treatment with PEF simultaneously induces the permeabilization of the whole cell barrier, both the cell wall and the membrane. This is responsible for the increase of the iron ions adsorption on the surfaces of negatively charged yeast cells and the interaction between it and the positive iron ions. The elevated amount of iron in cells caused, however, over 40% decrease in the fermentative activity of yeast as compared to the control sample. Overall iron-enriched yeast may be considered an additional source of this element in a diet.

## Figures and Tables

**Table 1:** The most important second derivative bands obtained in the spectra of all yeast samples, and the type of vibrations with assigned cellular components.

Wavenumber (cm^−1^)			Assignment of the Type of Vibrations
C1	C2	Fe + PEF	
2962	2958	2960	ν_as_(CH_3_) of lipids
2925	2925	2926	ν_as_(CH_2_) of lipids
2851	2856 *	2856 *	ν_s_(CH_2_) of lipids
1653	1654	1655	80% ν(C=O), 20% ν(C–N) of amide I, τ(HOH) of water
1635	1635	1637	
1540	1543 *	1543 *	60% τ(N–H), 30% ν(C–N), 10% ν(C–C) of amide II
1152	1152	1150	ν(C–O) of carbohydrates (mannans and β-1,3 glucans)
1080	1078	1078	ν_as_(PO_2_^−^) of membrane lipids, carbohydrates
1043	1047 *	1047 *	ν_s_(PO_2_^−^), mannans
1030	1025 *	1025 *	β-1,4 glucans
991	993 *	995 *	β-1,6 glucans

The symbols concerning the vibrations assignment are related to the stretching vibrational mode (ν), deformational (δ); bending (τ), and symmetrical (s) and asymmetrical (as) modes. An assignment of spectral features was collected according to the literature [41,42]. Asterisks (*) indicate the most significant spectral shifts (>2 cm^−1^) between the control sample (C1) and the iron-enriched samples both without PEF treatment (C2) and with the use of PEF (Fe + PEF).

**Table 2:** Protein content in yeast: C1—without the addition of iron ions and not subjected to PEF; C2—with the addition of iron ions (200 μg/mL) and not subjected to PEF, Fe + PEF—with the addition of iron ions (200 μg/mL) and subjected to PEF (ferric nitrate, voltage 1500 V, pulse width 10 µs, treatment time 20 min, 1200 pulses, after 20 h of cultivation). Each value is the mean ± standard deviation (*n* = 3). Means with the same letter (a–c) are not significantly different (*p* < 0.05).

Sample	Protein Content (%)	Iron Content (mg/g Dry Mass)
C1	59.13 ± 0.18 ^a^	0.13 ± 0.01 ^a^
C2	58.24 ± 0.35 ^b^	18.68 ± 0.86 ^b^
Fe + PEF	54.07 ± 0.11 ^c^	48.01 ± 0.88 ^c^

**Table 3:** The composition of media used in the experiment.

Medium for Inoculum Growth	Concentration (g/L)
Sucrose (POCH, Gliwice, Poland)	20
NH_4_Cl (POCH, Gliwice, Poland)	3.2
KH_2_PO_4_ (POCH, Gliwice, Poland)	2.5
Na_2_SO_4_ (POCH, Gliwice, Poland)	2.0
MgCl_2_•6H_2_O (POCH, Gliwice, Poland)	1.5
Yeast extract (BTL, Łódź, Poland)	5.0
Agar (DIFCO, Detroit, MI, USA)	15.0
Unhopped wort (Lublin Breweries S.A., Lublin, Poland)	40 mL
Experimental medium	
Peptone (Sigma–Aldrich CO, St. Louis, MO, USA)	10.0
Yeast extract (BTL, Łódź, Poland)	5.0
Glucose (POCH, Gliwice, Poland)	10.0

## Data Availability

Not applicable.

## Supplementary Materials

The following are available online at https://www.mdpi.com/article/10.3390/biom11060850/s1, Figure S1: ATR FTIR spectra of all yeast samples (*S. cerevisiae*) investigated acquired in the wavenumber range from 4000 to 750 cm^−1^.The most prominent absorbance bands are indicated and correspond to the stretching vibrations of CH_2_ and CH_3_ groups in lipids, amide I (~1640 cm^−1^) and amide II (~1530 cm^−1^) bands of proteins and to the carbohydrate region between 1200 and 900 cm^−1^. Each spectrum presented is an average of three independent measurements and normalized for equal area between 1180 and 900 cm^−1^, Table S1: The most important bands obtained in the spectra of yeast, and the type of vibrations with assigned sample components.
