# Supplementary material for: Pulsed Electric Field (PEF) Enhances Iron Uptake by the Yeast Saccharomyces cerevisiae
Source: Biomolecules. 2021 Jun 7;11(6):850. doi: 10.3390/biom11060850 (PMC8227778; doi:10.3390/biom11060850)
Supplement: Supplementary file 1 [file biomolecules-11-00850-s001.zip › biomolecules-1223952-supplementary.pdf]

Supplementary Materials

Pulsed Electric Field (PEF) Enhances Iron Uptake by the Yeast *Saccharomyces cerevisiae*

Karolina Nowosad [1], Monika Sujka [1,*], Urszula Pankiewicz [1], Damijan Miklavčič [2] and Marta Arczewska [3]

[1] Department of Analysis and Evaluation of Food Quality, Faculty of Food Sciences and Biotechnology,
University of Life Sciences in Lublin, Skromna 8, 20-704 Lublin, Poland;
karo.nowosad@gmail.com (K.N.); urszula.pankiewicz@up.lublin.pl (U.P.)

[2] Faculty of Electrical Engineering, University of Ljubljana, Trzaska cesta 25, 1000 Ljubljana, Slovenia;
damijan.miklavcic@fe.uni-lj.si

[3] Department of Biophysics, Faculty of Environmental Biology, University of Life Sciences in Lublin,
Akademicka 13, 20-950 Lublin, Poland; marta.arczewska@up.lublin.pl

* Correspondence: monika.sujka@up.lublin.pl; Tel.: +48-81-462-3333

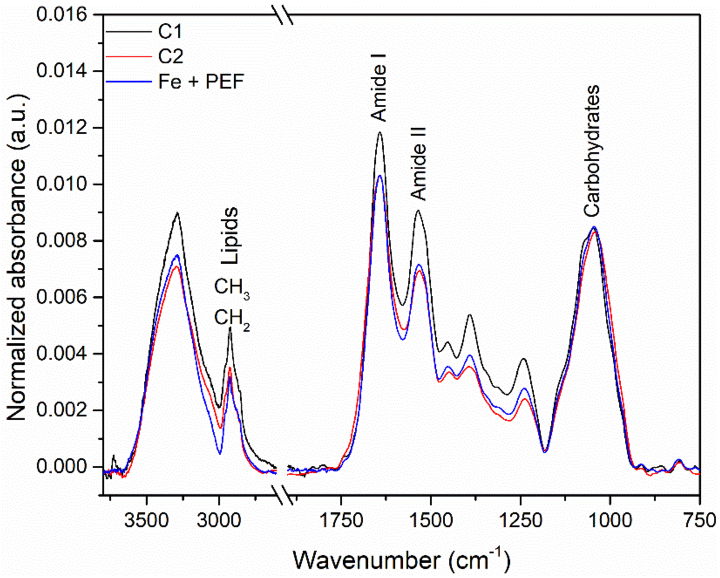

Figure S1. ATR FTIR spectra of all yeast samples (S. *cerevisiae*) investigated acquired in the wavenumber range from 4000 to 750 cm^{-1}.The most prominent absorbance bands are indicated and correspond to the stretching vibrations of CH_2 and CH_3 groups in lipids, amide I (~1640 cm^{-1}) and amide II (~1530 cm^{-1}) bands of proteins and to the carbohydrate region between 1200 and 900 cm^{-1}. Each spectrum presented is an average of three independent measurements and normalized for equal area between 1180 and 900 cm^{-1}.

Table S1. The most important bands obtained in the spectra of yeast, and the type of vibrations with assigned sample components.

Wavenumber [cm^{-1}]			Assignment of the type of vibrations
C1	C2	Fe+PEF	
3286	3291	3291	ν (N–H), ν (O-H) amide A, water
2956	2955	2955	ν_{as} (CH_3), lipids
2924	2924	2926	ν_{as} (CH_2), lipids
2853	2853	2855	ν_s (CH_2), lipids
1742	1741	1739	ν (C=O), phospholipids
1640	1642	1645	80% ν (CO), 20% ν (CN), τ (HOH), amide I, water
1534	1532	1532	60% τ (N–H), 30% ν (C–N), 10% ν (C–C), amide II
1452	1452	1451	δ_{as} (CH_3), δ_{as} (CH_2), proteins, lipids
1392	1392	1390	δ_s (CH_3), δ_s (CH_2), ν_s (C=O), proteins, lipids
1240	1237	1237	ν_{as} (PO_2^-), phospholipids
1139	1130sh	1130sh	ν (C–O), carbohydrates
1070	1070sh	1070sh	ν (C–O), carbohydrates
1046	1045	1045	ν_s (PO_2^-), carbohydrates

[a]The symbols concerning the vibrations assignment are related to the stretching vibrational mode (ν), deformational (δ); bending (τ), and symmetrical (s) and asymmetrical (as) modes. An assignment of spectral features was collected according to the literature [1].

References

1. Oberg, K.A.; Ruysschaert, J.-M.; Goormaghtigh, E. The optimization of protein secondary structure determination with infrared and circular dichroism spectra. *Eur. J. Biochem.* **2004**, *271*, 2937–2948.
